# Supplementary material for: Small RNA GcvB Regulates Oxidative Stress Response of Escherichia coli
Source: Antioxidants (Basel). 2021 Nov 5;10(11):1774. doi: 10.3390/antiox10111774 (PMC8614746; doi:10.3390/antiox10111774)
Supplement: Supplementary file 1 [file antioxidants-10-01774-s001.zip › antioxidants-1441198-supplementary.pdf]

Supplementary information

Table S1. Strains used in this work

Strain	Genotype	Description
NCM3722	wild-type *E. coli* K-12 strain	wild-type *E. coli*K12 strain
CY325	△*gcvB*	*gcvB* deletion in NCM3722
CY713	wild-type *E. coli* K-12 strain	repaired NCM3722 with wild-type *fliC*
CY1027	△*gcvB::cat*	*gcvB* deletion in CY713
CY1038	△*oxyR::cat*	*oxyR* deletion in CY713
CY454	*oxyR-myc*-stop codon-*kan*	*oxyR-myc* constructed in NCM3722
CY455	*oxyR-myc*-stop codon-*kan* △*gcvB*	*oxyR-myc* constructed in CY325
CY1042	Δ*gcvB oxyR*::*cat*	*oxyR and gcvB* double deletion strain
CY1037	*kan*::P1*oxyR-lacZ* Δ*oxyR*::*cat*	P1*oxyR-lacZ* translational fusion constructed in the *gcvB* wild-type background CY1057
CY1041	kan::P1oxyR-lacZ Δ*oxyR*::*cat* Δ*gcvB*	P1*oxyR-lacZ* translational fusion constructed in the *gcvB* knockout background CY1042
CY1047	*kan*::P3*oxyR-lacZ* Δ*gcvB* Δ*oxyR*::*cat*	P3*oxyR-lacZ* transcriptional fusion constructed in the *gcvB* knockout background CY1042
CY1052	*kan*::P3*oxyR-lacZ* Δ*oxyR::cat*	P3*oxyR-lacZ* transcriptional fusion constructed in the *gcvB* wild-type background CY1057
CY1057	Δ*oxyR*::*cat*	*oxyR* deletion in NCM3722 background
CY1059	*kan*::P2*oxyR-lacZ* Δ*oxyR*::cat	P2*oxyR-lacZ* translational fusion constructed in the *gcvB* wild-type background CY1057
CY1060	*kan*::P2*oxyR-lacZ* Δ*oxyR*::cat Δ*gcvB*	P2*oxyR-lacZ* translational fusion constructed in *gcvB* knockout background CY1042

Table S2. Primers used for RT-qPCR

Primers	Sequences
flu-F	AACCGGGACAGTGATGAGAG
flu-R	AGACGGACGCTGTTGTTTTC
yeeR-F	TCATGAAACTGCCGGAAACC
yeeR-R	GGATTTCTGTTCCGCTGACC
oxyR-F	CTGATGCTGGAAGATGGTCA
oxyR-R	AGCGCTGGCAGTAAAGTGAT
recA-F	GTGCGTTTATCGATGCTGAAC
recA-R	CACAGATTTCCAGTGCCTGCT

Table S3. DEGs identified between the *gcvB* knockout and wild-type strains.

ID	Symbol	EXP_{WT}	EXP_{CY325}	Log2(FC)	*q*-value (FDR)
AA953_RS06745	*fhuC*	329.9075	142.12	-1.23433	7.49E-08
AA953_RS06750	*fhuD*	304.025	134.6325	-1.2105	1.04E-07
AA953_RS06755	*fhuB*	156.0425	77.0725	-1.05673	0.000417
AA953_RS16720	*flu*	44.44	2769.188	5.985994	2.09E-46
AA953_RS16725	*yeeR*	23.0725	104.1275	2.201021	7.12E-06

DEGs: differentially expressed genes

EXP_{WT} and EXP_{CY325} respectively indicate gene expression (FPKM) in wild-type NCM3722 and the *gcvB* deletion strain CY325.

FC: Fold change.

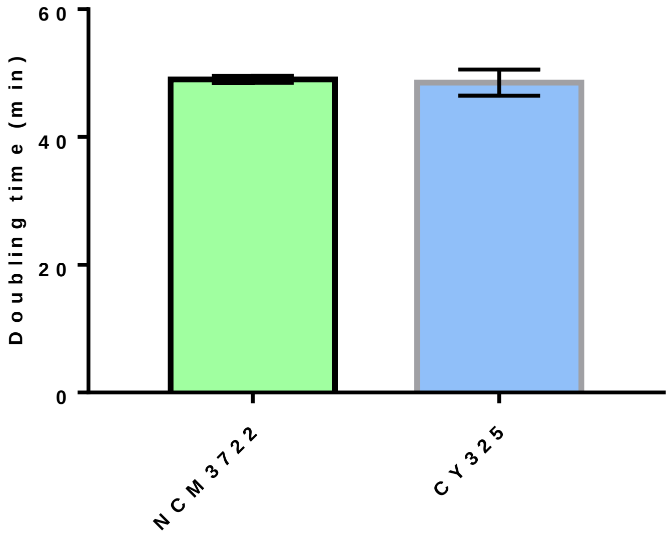

Figure S1. Doubling time of NCM3722 (WT) and CY325 (△*gcvB*) grown in glucose minimal medium.

Doubling time of each strain is expressed as average ± S.D. of three independent cultures.

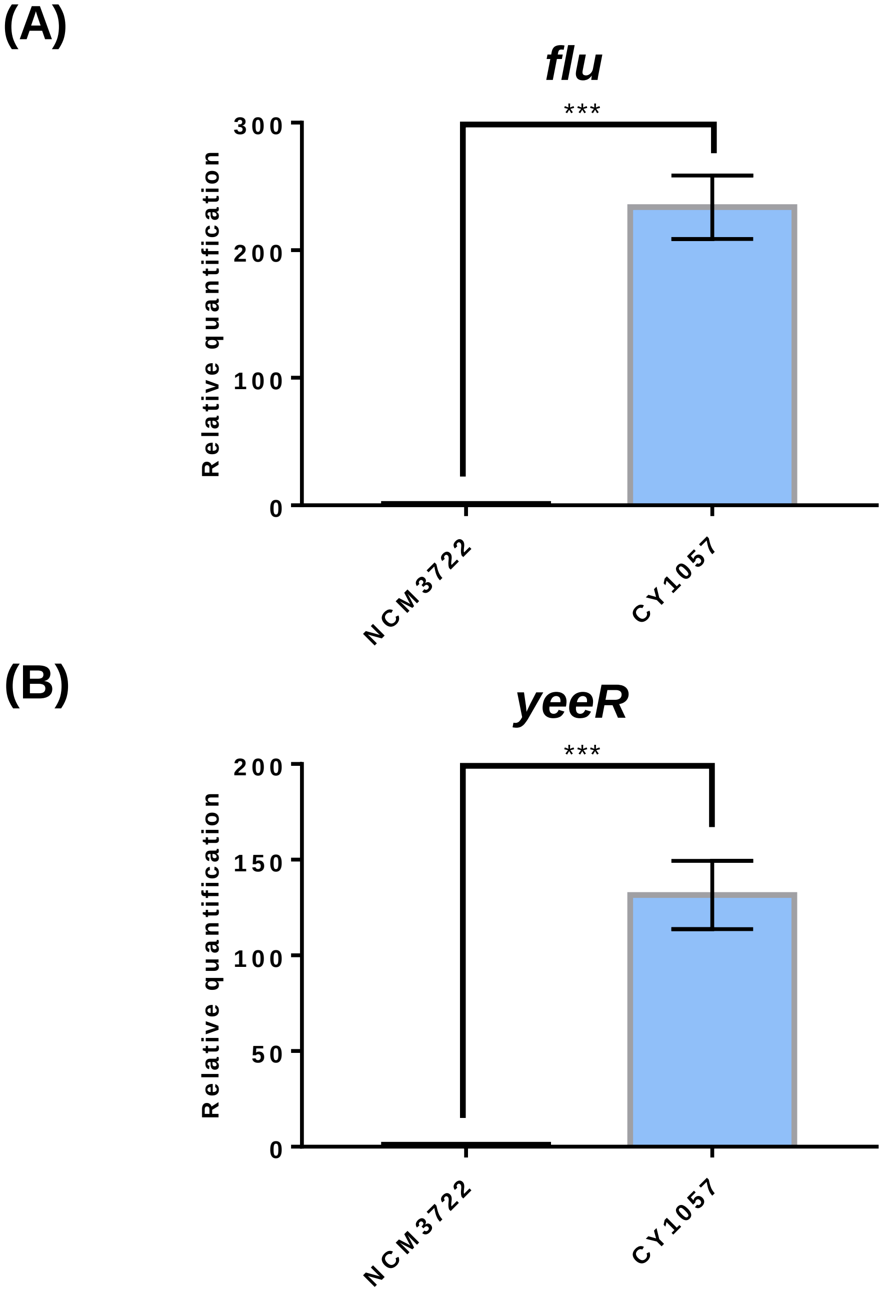

Figure S2. Gene expression of *flu* and *yeeR* in the *oxyR* wild-type and knockout strains. A-B. Gene expression of *flu* and *yeeR* detected by RT-qPCR in the *oxyR* knockout strain CY1057 and in the wild-type strain NCM3722 grown in glucose minimal medium. The mRNA level of each gene in NCM3722 was normalized to 1 and that in CY1057 was determined relative to this value. The relative expression was shown as the average ± S.D. of three independent experiments. ***$P < 0.001$ by student t-test.

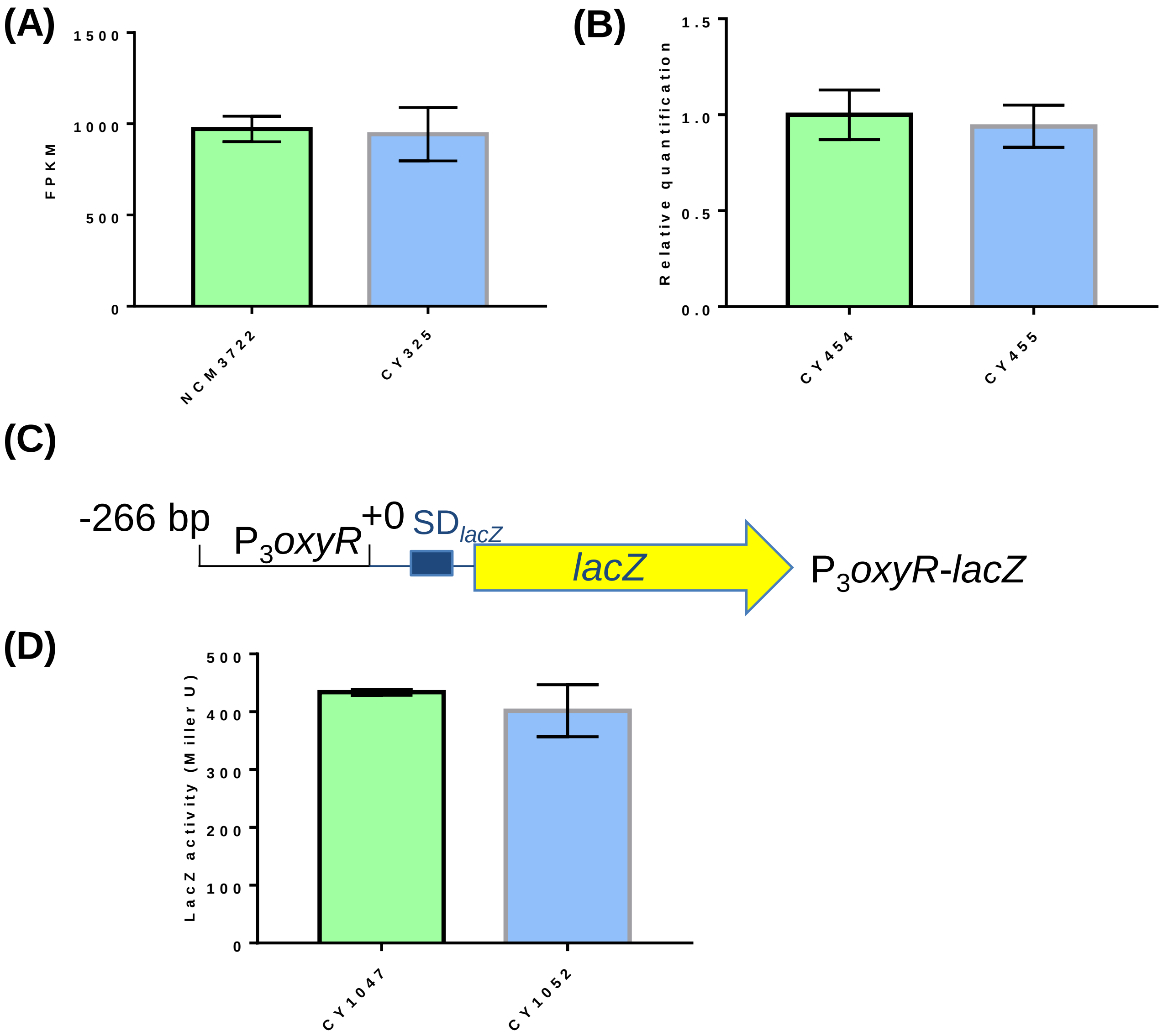

Figure S3. Transcriptional expression of *oxyR*.
A. The FPKM values of *oxyR* expression in the wild-type strain NCM3722 and in the *gcvB* knockout strain CY325 obtained by RNAseq. The FPKM values are shown as the average ± S.D. of two RNAseq assays. **B.** Gene expression of *oxyR* detected by RT-qPCR in the *gcvB* knockout (CY455) and wild-type (CY454) strains grown in glucose minimal medium. The mRNA level of each gene in CY454 was normalized to 1 and that in CY455 was determined relative to this value. The relative expression was shown as the average ± S.D. of three independent experiments. **C.** Composition diagram of the promoter of *oxyR* with *lacZ* transcriptional fusion. P_3*oxyR* covers the DNA region from -266 bp to 0 bp relative to *oxyR* translational start point. SD_{lacZ} indicates the ribosome binding site of *lacZ*. Yellow arrow indicates the entire coding region of *lacZ*. **D.** β-galactosidase activities of P_3*oxyR-lacZ* fusion in the *gcvB* wild-type (CY1052) and knockout strains (CY1047).The LacZ activity was shown as the average ± S.D. of three independent experiments.
